# Polar amplification comparison among Earth’s three poles under different socioeconomic scenarios from CMIP6 surface air temperature

**DOI:** 10.1038/s41598-022-21060-3

**Published:** 2022-10-03

**Authors:** Aihong Xie, Jiangping Zhu, Shichang Kang, Xiang Qin, Bing Xu, Yicheng Wang

**Affiliations:** 1 State Key Laboratory of Cryospheric Sciences, Northwest Institute of Eco-Environment and Resources, Chinese Academy of Sciences, Lanzhou, China; 2 University of Chinese Academy of Sciences, Beijing, China; 3 Lanzhou Central Meteorological Observatory, Lanzhou, China

**Keywords:** Projection and prediction, Cryospheric science

## Abstract

The polar amplification (PA) has become the focus of climate change. However, there are seldom comparisons of amplification among Earth’s three poles of Arctic (latitude higher than 60 °N), Antarctica (Antarctic Ice Sheet) and the Third Pole (the High Mountain Asia with the elevation higher than 4000 m) under different socioeconomic scenarios. Based on CMIP6 multi-model ensemble, two types of PA index (PAI) have been defined to quantify the PA intensity and variations, and PAI1/PAI2 is defined as the ratio of the absolute value of surface air temperature linear trend over Earth’s three poles and that for global mean/over other regions except Earth’s three poles. Arctic warms fastest in winter and weakest in summer, followed by the Third Pole, and Antarctica warms least. The similar phenomenon proceeds when global warming of 1.5–2.0 °C, and 2.0–3.0 °C above pre-industrial levels. After removing the Earth’s three poles self-influence, all the PAI2s increase much more obviously relative to the PAI1s, especially the Antarctic PAI. Earth’s three poles warm faster than the other regions. With the forcing increasing, PA accelerates much more over Antarctica and the Third Pole, but becomes weaker over Arctic. This demonstrates that future warming rate might make a large difference among Earth’s three poles under different scenarios.

## Introduction

With the introduction of the Third Pole^1^, the Arctic, Antarctica and the Third Pole have been collectively referred to as the “three poles of the Earth”^2^, and become a new focus of climate change research^3, 4^. Earth’s three poles are sensitive to climate change^2^, and also play an important role in climate system^5, 6^. The cryosphere melting from Earth’s three poles makes an outstanding contribution to sea-level rise with the glacier, snow cover, sea ice and ice sheet experiencing a widespread and conspicuous shrinking during the last decades^5, 7–10^.

Surface air temperature (SAT) plays a vital role in the cryosphere melting of Earth’s three poles with the unprecedented global warming^9, 11^. Each of the last four decades has been successively warmer than any decade since 1850^11^. Global SAT was 0.99 °C higher in 2001–2020, and 1.09 °C higher in 2011–2020 than 1850–1900, with larger increases over land (1.59 °C) than over ocean (0.88 °C)^11^. Earth’s three poles exhibit the different temperature variations^2^. The Arctic region including Arctic Ocean has warmed two to four times as fast as the global average over the last few decades, commonly known as Arctic amplification (AA)^8, 12, 13^. Different from the Arctic homogeneous warming, Antarctic temperature change is more complicated, with the general characteristic of faster warming in Antarctic Peninsula and West Antarctica than in East Antarctica^14, 15^ and even with the regional warming absent^16, 17^. The strong warming in high-elevation regions is an intrinsic feature of recent global warming^18^, and the warming magnitude over the Third Pole is smaller than that over high latitudes in the northern hemisphere^19, 20^, but larger than Southern Hemisphere and tropics^21^.

The conspicuous increase of Greenland Ice Sheet melting and its contribution to sea level rise in the future is largely determined by the intensity of Arctic amplification and the global equilibrium climate sensitivity^10, 22, 23^. As a robust feature of polar warming seen in historical observations and climate model simulations, polar amplification has been doubtful for the mechanism. Besides the natural climate variability, polar amplification in recent decades mainly contributes to the positive local lapse-rate feedback^24^, including ice-albedo and Planck feedbacks acting as supporting roles^25^, downward clear-sky longwave feedbacks, and surface albedo feedback^2, 26^, etc. Accurate predictions of polar warming are critical given the fundamental role that polar ice plays in climate system, terrestrial and marine ecosystems, and human society^2, 7^. However, comparison research on the amplification of Earth’s three poles has still been scarce. Therefore, our goal is (1) to compare the amplification variation of Earth’s three poles during 2015–2100, with the CMIP6 multi-model outputs under different socioeconomic pathways (SSPs) of SSP1-1.9, SSP1-2.6, SSP2-4.5, SSP3-7.0 and SSP5-8.5, and under global warming of 1.5 °C, 2.0 °C and 3.0 °C above pre-industrial levels; (2) to quantify and compare the pole amplification with other regions during 2015–2100 through different polar amplification indices (PAIs).

## Data and methods

### Data sources

The Coupled Model Intercomparison Project (CMIP) works to better understand the past, present and future climate change caused by natural and unforced variability^27^. In the CMIP6 latest-generation climate models, one third exhibits the higher equilibrium climate sensitivity^28^, and a new set of alternative pathways of future societal development are described as shared socioeconomic pathways (SSPs) of future radiative forcing used for sustainable development problems analysis^29^. Forcings are provided by the integrated assessment model for the future scenarios in Scenario Model Intercomparison Project (ScenarioMIP), harmonized with atmospheric emissions, concentrations and land use^57^. The ScenarioMIP climate models from CMIP6 under the five SSPs of SSP1-1.9, SSP1-2.6, SSP2-4.5, SSP3-7.0, and SSP5-8.5 that cover the range of possible future development of anthropogenic drivers of climate change found in the literature^11^, and the five socioeconomic scenarios are used to assess the surface air temperature change of Earth’s three poles in twenty-first century. For the limit of CMIP6 model outputs in SSP1-1.9, we only use seven models (Table S3) to analyze the climate change during the period 2015–2100. The model outputs from the CMIP6 “historical” experiments cover the period 1850–2014, and are driven by observed boundary conditions^30^.

### Study area

**Figure 1:**
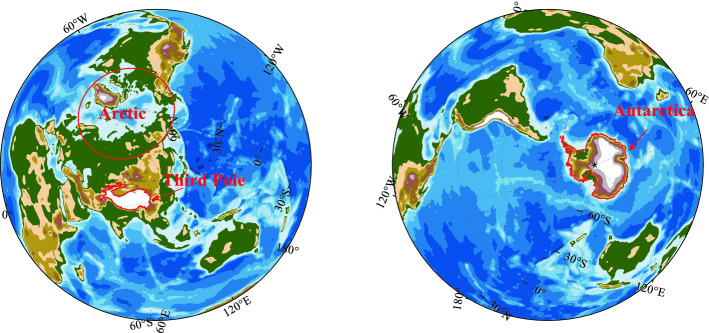
The locations of the Earth’s three poles. The map was drawn in the software MATLAB R2018b (https://ww2.mathworks.cn/products/matlab.html).

This study focuses on the different extent for the three poles, and the locations of the three poles are shown in Fig. 1. The Arctic refers to the region that latitude higher than 60 °N^5^, Antarctica refers to the Antarctic Ice Sheet, and the Third Pole is the High Mountain Asia with the average elevation more than 4000 m, including the highest and most spatially extensive highland in the world^27, 31^.

### Definition of amplification index

Polar amplification is commonly defined as the ratio of polar warming to tropical warming, which can estimate the robust feature of climate change^25^. To quantitate the amplification over Earth’s three poles, the polar amplification index (PAI) has been defined as the ratio of the absolute value of surface atmosphere temperature (SAT) linear trend over Earth’s three poles and that for global mean (PAI1). To eliminate the self-influence from the loop computation of the three poles, this study has also defined one other PAI as the ratio of the SAT trend over Earth’s three poles and that over other regions except Earth’s three poles (PAI2), to further compare the warming between Earth’s three poles and other regions.

### Calculation from CMIP6 data

**Figure 2:**
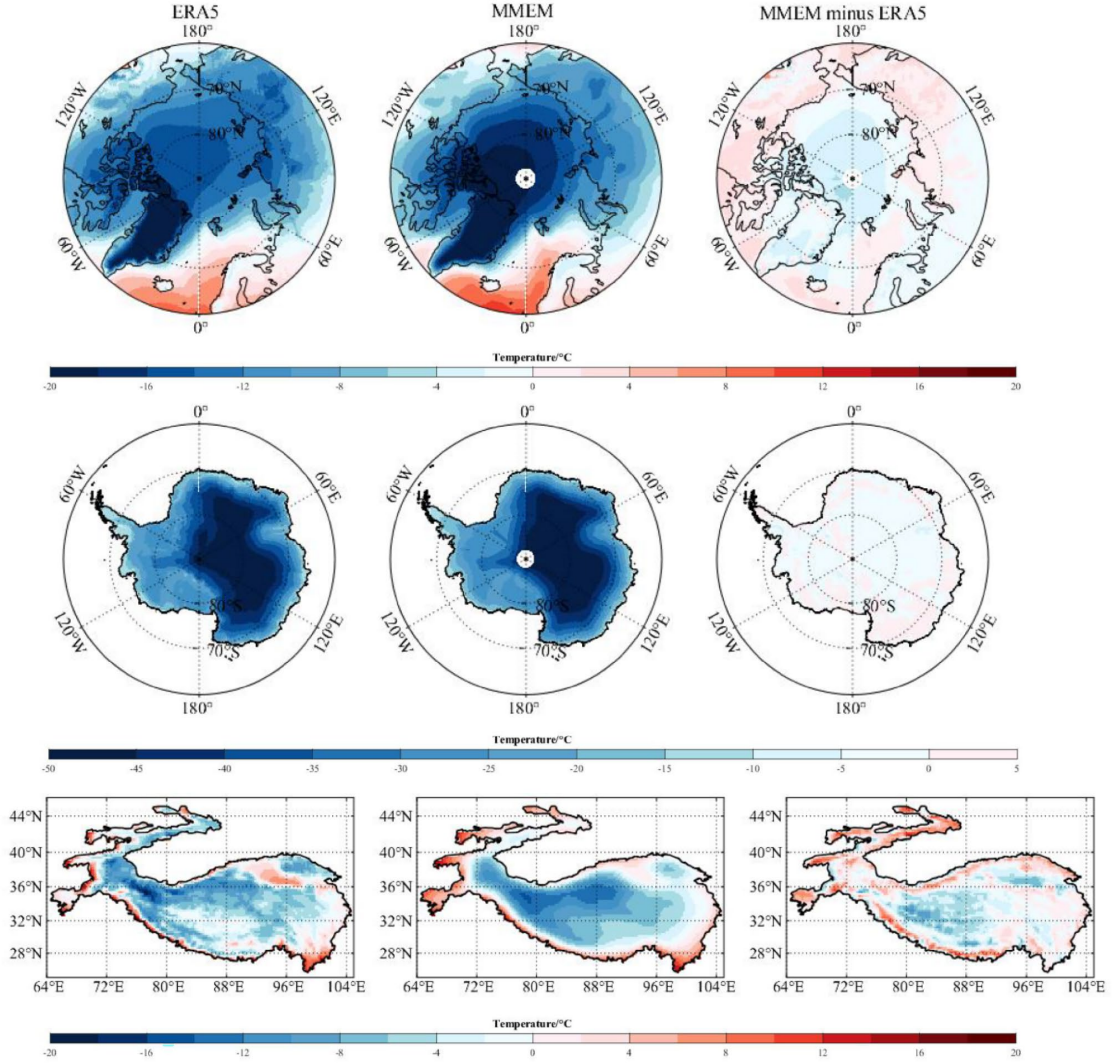
Spatial patterns of annual mean temperature from the ERA5 (left panel), the multi-model ensemble mean (MMEM) of the CMIP6 model simulations (center panel), and their biases (MMEM minus ERA5) (right panel) over the Arctic, Antarctica and The Third Pole during 1979–2014. The map was drawn in the software MATLAB R2018b (https://ww2.mathworks.cn/products/matlab.html).

To uniform the resolution from different models, the scenario data are regridded into the resolution of 0.25° × 0.25° using the bilinear interpolation. With considering uncertainties, the multi-model ensemble mean (MMEM) is an appropriate way for the model outputs, which weighs all models equally^20, 32^. ERA5 is the latest atmospheric reanalysis produced by European Centre for Medium-Range Weather Forecasts (ECMWF), and it has high skill in representing the temperature across the Earth’s three poles^33–35^. Generally, temperature from the MMEM can capture the spatial patterns over the Arctic, Antarctica and the Third Pole, although the bias inevitably exists (Fig. 2).

We have employed the simple linear regression to calculate the trend of temperature, and the regression procedure chooses the line producing the least error^36^, and F test is used to estimate the significance (*p* < 0.05) of temperature trends. For the regional mean, the temperature is calculated by simply averaging the grid data in the area, and further obtain the regional trend and calculate the regional PAI. Similarly, the spatial analysis of trend is based on a simple regression of the temperature at each grid point. The spatial distribution of the PAI1/PAI2 is the absolute ratio of the grid temperature trend to the global average temperature trend/the trend over the other regions except Earth’s three poles.

## Results

### Amplification comparison among Earth’s three poles under different socioeconomic scenarios

**Figure 3:**
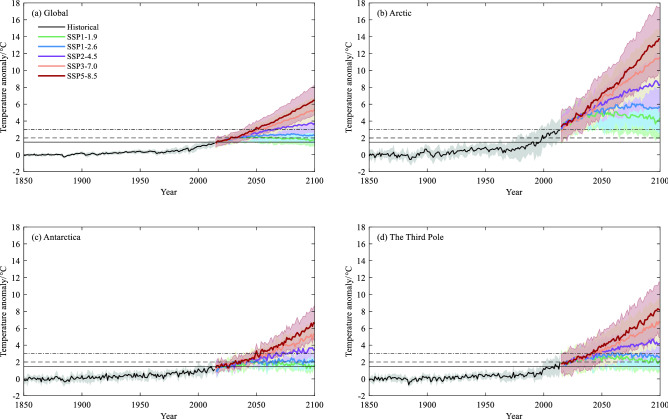
Annual mean surface air temperature anomaly (°C, 1850–1900 as the baseline period) for (**a**) Global, (**b**) Arctic, (**c**) Antarctica and (**d**) The Third Pole during 1850–2100 from multi-model ensemble mean (MMEM) of the CMIP6 simulations under the historical (black curve), SSP1-1.9 (green), SSP1-2.6 (blue), SSP2-4.5 (purple), SSP3-7.0 (orange), SSP5-8.5 (bronze/crimson), respectively. The black straight lines are for 1.5 °C (solid line), 2.0 °C (break line), 3.0 °C (breakpoint line), respectively.

**Figure 4:**
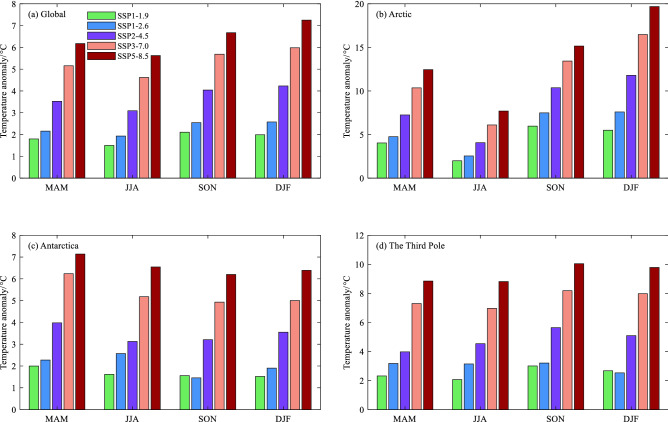
Surface air temperature anomaly (°C,1850–1900 as the baseline period) for seasonal mean for (**a**) Global, (**b**) Arctic, (**c**) Antarctica and (**d**) The Third Pole in 2100 from multi-model ensemble mean (MMEM) of the CMIP6 simulations under SSP1-1.9 (green), SSP1-2.6 (blue), SSP2-4.5 (purple), SSP3-7.0 (orange), SSP5-8.5 (bronze), respectively. The season mean is from the three monthly average of MAM (March–May), JJA (June–August), SON (September–November) and DJF (December-February), respectively.

The time series in Fig. 3 shows the annual surface air temperature anomaly relative to the 1850–1900 baseline period over the Arctic, Antarctica, the Third Pole and global mean during the period 1850–2100 from MMEM under different SSPs of SSP1-1.9, SSP1-2.6, SSP2-4.5, SSP3-7.0 and SSP5-8.5. The annual temperature over the Arctic and the Third Pole obviously increases faster than global mean, especially in the high forcing socioeconomic scenarios. Under SSP5-8.5, the global mean will rise by approximately 6.0 °C at the end of twenty-first century, and the increase over the Arctic and the Third Pole are higher than 8.0 °C, which indicates the occurrence of amplification and is consistent with the previous research^37, 38^, whereas the signal is weak in Antarctica. The Arctic warming is most conspicuous among Earth’s three poles. Even under SSP1-1.9 socioeconomic scenario, Arctic warms more than 3.0 °C. However, the Antarctic temperature rises lower than 2.0 °C in the SSPs of SSP1-1.9 and SSP1-2.6. Similar to annual mean, the three poles exhibit different sensitivity to future forcing. Global mean temperature shows the strong warming in DJF (December-February) except under SSP1-1.9 (Fig. 4). Under SSP3-7.0 and SSP5-8.5, Arctic will experience further warming of at least 17 °C in DJF compared with the 1850–1900 baseline by the end of the twenty-first century. Similarly, the greatest seasonal variability of Antarctic temperature occurs in winter JJA (June–August), with the warming anomaly of 6.34 °C under SSP5-8.5. Projections show that the most conspicuous warming of the Third Pole occurs in SON (September–November), ranging from 3.00 °C under SSP1-1.9 to 10.04 °C under SSP5-8.5 in 2100 relative to the baseline period.

**Figure 5:**
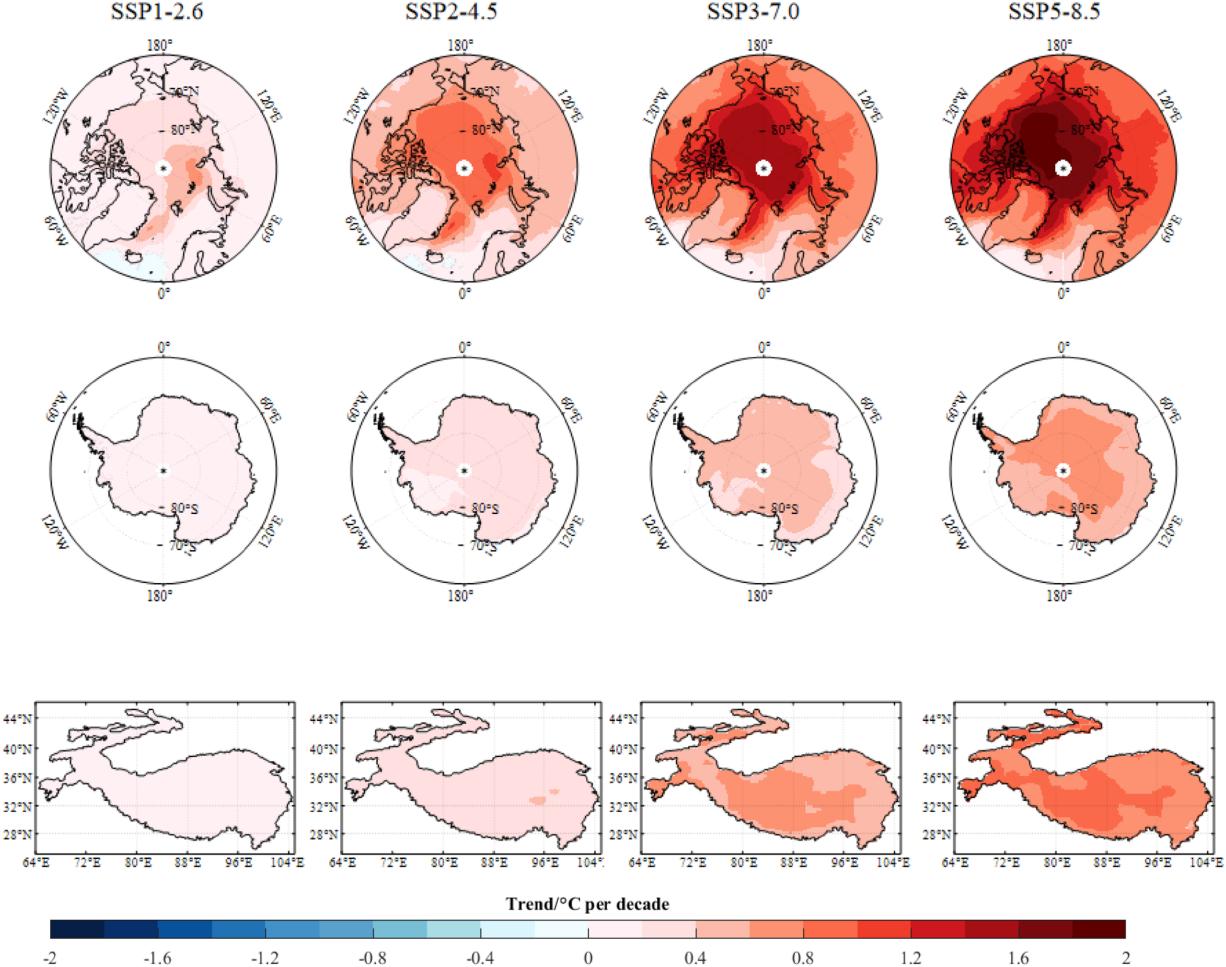
Spatial patterns of annual temperature trend over the Arctic, Antarctica and The Third Pole from the multi-model ensemble mean (MMEM) of the CMIP6 simulations under different SSPs during 2015–2100. The grey dotted region represents that the trend is not significant. The map was drawn in the software MATLAB R2018b (https://ww2.mathworks.cn/products/matlab.html).

The distribution of annual temperature trend under different SSPs over Earth’s three poles during the period 2015–2100 is shown in Fig. 5. Obviously, the strongest warming occurs over the Arctic in all SSPs. Over large parts of the Arctic, annual mean temperatures increase higher than 1.0 °C per decade for the high-emission SSPs of SSP3-7.0 and SSP5-8.5. The conspicuous Arctic warming exceeds 2.0 °C per decade in DJF (not shown). Antarctic temperature shows the significantly positive trends over almost all grids, while the trends are generally weaker than Arctic and the Third Pole. MMEM reveals conspicuous warming in JJA, especially over the Antarctic coast region. Over the Third Pole, faster warming concentrates on the northern Tibet Plateau under high-emission SSPs of SSP3-7.0 and SSP5-8.5. The strongest changing sign occurs in autumn over the Third Pole, with the warming above 1.0 °C per decade in SSP5-8.5. Among Earth’s three poles, there is a broad consistency of warming signal in the future, with the strongest positive trends in Arctic and weakest in Antarctica.

**Figure 6:**
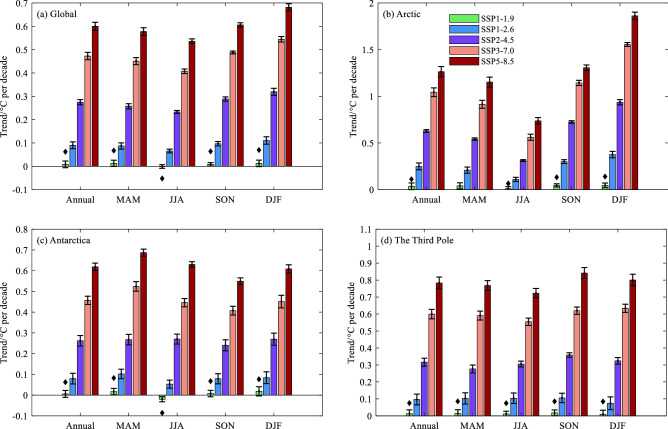
Similar to Fig. 4, but for regional average trends (°C per decade) during 2015–2100. The black rhombus symbol ♦ represents that the trend is not significant. The others are significant at the 95% confidence interval.

To further explore the temperature changes over Earth’s three poles, we calculate the trend of regional average for surface seasonal and annual temperature mean during 2015–2100 (Fig. 6). In all SSPs, Arctic performs the greatest increases, and the largest positive trend in DJF, with the rate of 0.05 °C, 0.37 °C, 0.94 °C, 1.56 °C and 1.86 °C per decade under SSPs of SSP1-1.9, SSP1-2.6, SSP2-4.5, SSP3-7.0 and SSP5-8.5, respectively. However, the Antarctic warming is rather weak with the trend of almost below 0.69 °C per decade, and even a negative trend occurs in austral winter JJA under SSP1-1.9. Over the Third Pole, conspicuous warming trend appears in SON, with the rate ranging from 0.02 °C under SSP1-1.9 to 0.97 °C under SSP5-8.5.

### Amplification comparison among Earth’s three poles under the global warming of 1.5 °C, 2.0 °C and 3.0 °C

The Paris Agreement of the United Nations Framework Convention on Climate Change (UNFCCC) sets a goal to limit the increase of global mean temperature to “well below 2 °C" and fights to limit warming to 1.5 °C relative to pre-industrial level^39^. Many researches aim to compare the changes under the global warming of 1.5 °C, 2.0 °C and 3.0 °C above pre-industrial levels, and/or examine whether a lower target warming is necessary^27, 40^. Global warming of 1.5 °C and 2.0 °C period is defined to be the time when the global average surface air temperature anomaly is 1.3–1.7°C and 1.8–2.2°C warmer relative to the pre-industrial period (1850–1900), respectively^27, 41^. To explore the temperature changes of Earth’s three poles in different thresholds, we calculate the regional temperature changes when global warming is from 1.5 °C to 2.0 °C. Under SSP1-1.9 the 2.0 °C warming is not possible and is omitted there.

When global mean warms from the 1.5 °C to 2.0 °C threshold, the increase of Arctic temperature is higher than 0.5 °C except in summer JJA, and annual mean warms 1.07 °C, 0.85 °C, 0.92 °C and 1.08 °C in SSP1-2.6, SSP2-4.5, SSP3-7.0 and SSP5-8.5 (Table S1), respectively. The change of Arctic temperature is conspicuous in autumn and winter, with the increase of 1.47 °C, 1.18 °C, 1.21 °C and 1.43 °C in SON from the lower forcing to the highest, and the corresponding value in DJF is 1.54 °C, 1.26 °C, 1.25 °C and 1.42 °C, respectively. Not surprisingly, Antarctica exhibits the slowest warming among Earth’s three poles, and even though Antarctic fails to warm 0.50 °C in austral winter JJA. For the Third Pole, annual mean successfully warms faster than global in all SSPs, and the strong warming signal occurs in autumn and winter.

**Figure 7:**
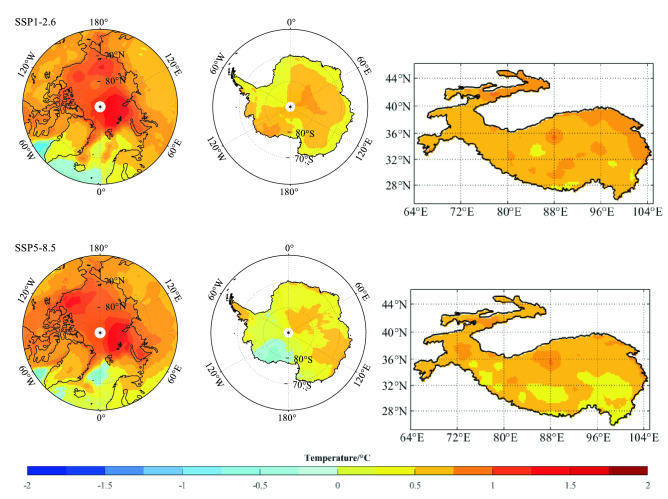
Spatial patterns of annual mean temperature difference (°C) over Arctic, Antarctica and The Third Pole from the CMIP6 simulations MMEM when global warming reaches 1.5 °C and 2.0 °C threshold above pre-industrial levels only under SSP1-2.6 and SSP5-8.5. The map was drawn in the software MATLAB R2018b (https://ww2.mathworks.cn/products/matlab.html).

When global mean warms from the 1.5 °C to 2.0 °C threshold, the annual warming of Earth’s three poles is obvious in SSP1-2.6 and SSP5-8.5 in general (Table S1), and these SSPs also represent the extreme forcing. Therefore, we only illustrate the distribution of annual temperature changing when global warming by 0.50 °C under these SSPs (Fig. 7). Clearly, most of Arctic regions warm faster than global, even some region warms more than twice as fast as the global average. The annual warming higher than 0.5 °C concentrates on the interior of East Antarctica in SSP1-2.6, and the most profound warming occurs in DJF. With the similar character in Arctic, the increase of the Third Pole temperature is also conspicuous in SON and DJF. Compared with Arctic, faster warming in the Third Pole can be found in JJA, with the increase of 0.82 °C, 0.50 °C, 0.49 °C and 0.79 °C under SSP1-2.6, SSP2-4.5, SSP3-7.0 and SSP5-8.5.

When global mean warms from 2.0 °C to 3.0 °C, the annual temperature mean over the Antarctica fails to achieve the 1.0 °C warming under the SSPs of SSP2-4.5, SSP3-7.0 and SSP5-8.5 (Table S2). In stark contrast, the strong warming of Arctic and the Third Pole can be observed, with the rise of annual mean exceeds 1.3 °C. For Arctic, the conspicuous warming occurs in DJF, with the increase of 3.65 °C, 3.93 °C and 3.45 °C from middle to the highest forcing. For the Third Pole, the changes of temperature exaggerate with the forcing increasing except in summer JJA.

### Amplification Quantification over Earth’s three poles through PAI1 under different socioeconomic scenarios

**Figure 8:**
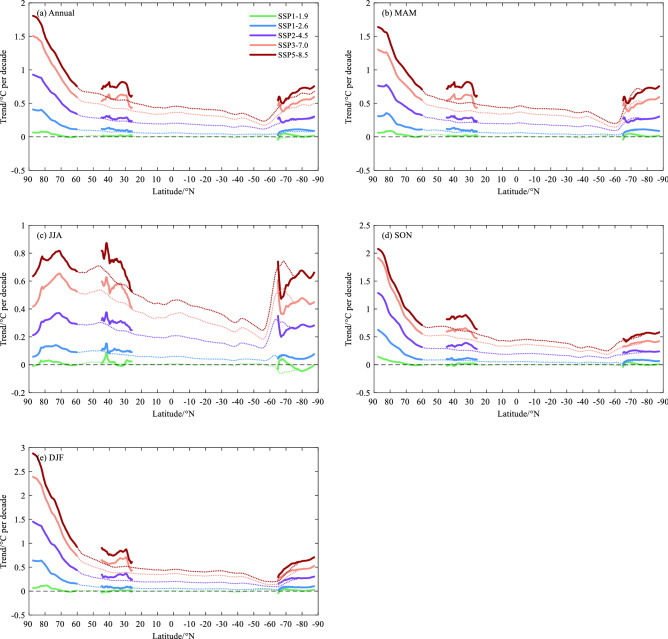
Zonally average temperature trends (°C per decade) for (**a**) annual and (**b–e**) seasonal during 2015–2100 from the CMIP6 simulations MMEM under the SSPs of SSP1-1.9, SSP1-2.6, SSP2-4.5, SSP3-7.0, SSP5-8.5, respectively. The dotted line is for the zonal average from latitude circles, and the solid is for the three poles.

The zonal mean temperature trends averaged from latitude circles for annual and seasonal mean under different SSPs is shown in Fig. 8. Inevitably, the strong AA can be captured in all cases. The zonal mean trend increases poleward to Arctic, with exception in JJA, and the phenomenon is symmetry in Antarctica. Generally, the zonal trend over Antarctica is higher than that of middle and low latitudes in southern hemisphere. However, the latitude effect is weaker than northern hemisphere. The meridional average trend only for the Third Pole region presents the faster change than the zonal mean, and it indicates the appearance of Third Pole amplification (TA).

Compared with the global mean, Earth’s three poles amplification index (PAI1) has been provided in Table 1, as the radio of the absolute value of SAT linear trend over Earth’s three poles and that for global mean. Table 1 quantifies the annual and seasonal variations in different SSPs, but excludes SSP1-1.9 socioeconomic scenario, since the SSP1-1.9 mean trend is too low to cause the index incredibility. Arctic amplification always exists with the annual warming PAI1 of 2.76, 2.29, 2.21 and 2.11 times than global mean under SSP1-2.6, SSP2-4.5, SSP3-7.0 and SSP5-8.5, respectively. In accordance with the annual mean rapid warming, the AA is most conspicuous in DJF, with the AA index of 3.39, 2.93, 2.86 and 2.73 under SSP1-2.6, SSP2-4.5, SSP3-7.0 and SSP5-8.5, respectively. However, Table 1 illustrates that Arctic Amplification might decelerate with the increase of radiative forcing from the low to high SSPs, except in summer JJA. Much weaker than AA, annual AnA doesn’t exist with the annual PAI1 lower than 1 except in SSP5-8.5. AnA also displays the seasonal variations, with all the PAI1 higher than 1 only in autumn MAM, and all the PAI1 lower than 1 in spring SON and summer DJF. Different from AA, TA enhances with the increase of forcing from the low to high socioeconomic scenarios. Much larger than AnA, TA dominates the whole year, especially in JJA and SON, with the amplification index higher than 1.20 in general.

**Table 1:** The Amplification Index (PAI1) based on global mean over Arctic, Antarctica, the Third Pole under SSP1-2.6, SSP2-4.5, SSP3-7.0 and SSP5-8.5, respectively.

	Annual	MAM	JJA	SON	DJF
**Arctic**					
SSP1-2.6	2.76	2.40	1.68	3.11	3.39
SSP2-4.5	2.29	2.10	1.34	2.51	2.93
SSP3-7.0	2.21	2.03	1.38	2.34	2.86
SSP5-8.5	2.11	1.99	1.38	2.16	2.73
**Antarctica**					
SSP1-2.6	0.88	1.17	0.81	0.82	0.76
SSP2-4.5	0.95	1.04	1.16	0.83	0.84
SSP3-7.0	0.97	1.16	1.09	0.83	0.83
SSP5-8.5	1.03	1.19	1.18	0.91	0.89
**The Third Pole**					
SSP1-2.6	1.07	1.18	1.58	1.09	0.67
SSP2-4.5	1.15	1.07	1.31	1.24	1.01
SSP3-7.0	1.27	1.31	1.36	1.27	1.16
SSP5-8.5	1.31	1.33	1.35	1.39	1.18

**Figure 9:**
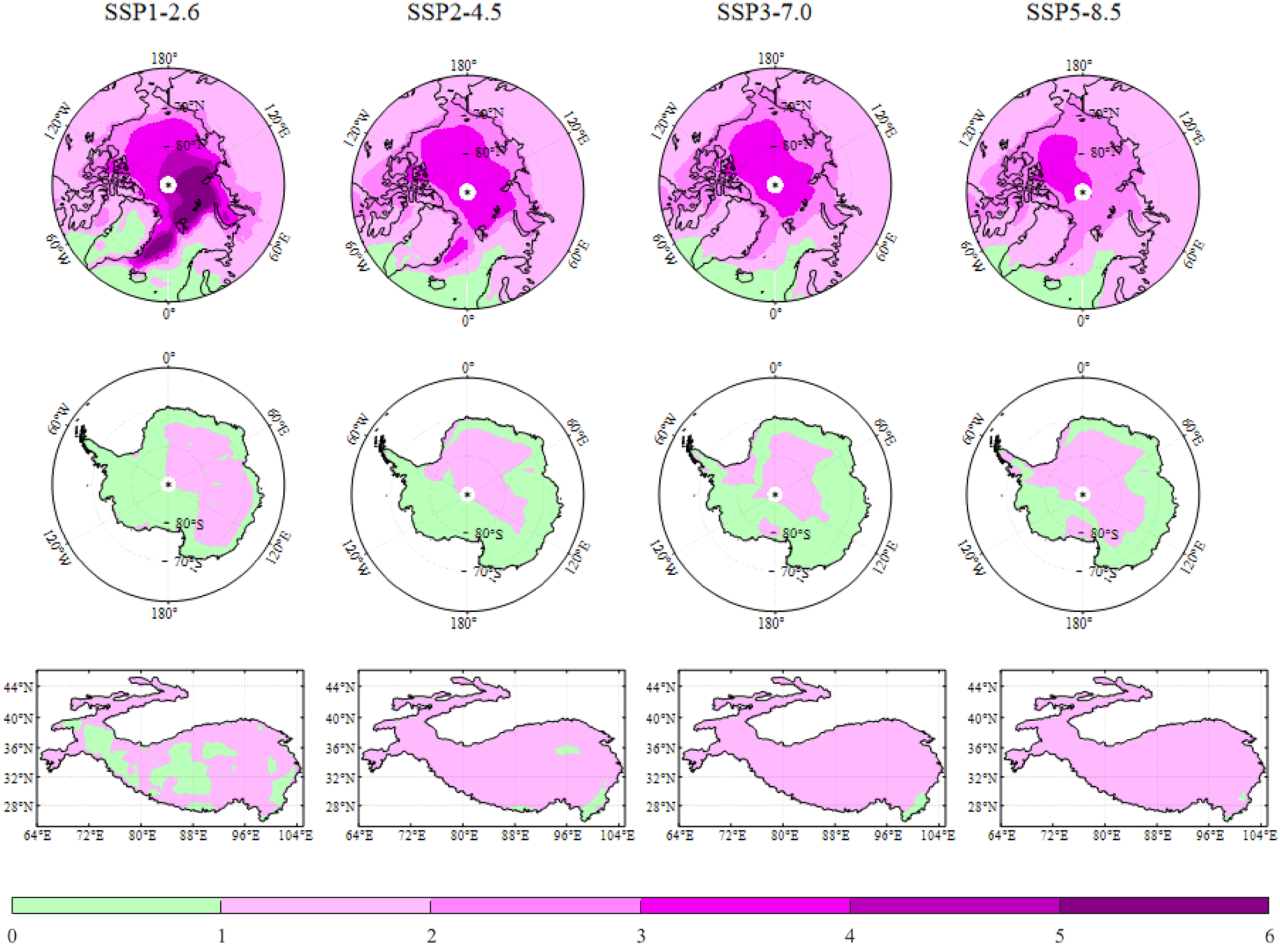
Similar to Fig. 5, but for the Polar amplification Index (PAI1) during 2015–2100. The map was drawn in the software MATLAB R2018b (https://ww2.mathworks.cn/products/matlab.html).

To better understand the amplification in Earth’s three poles, spatial patterns of amplification index for annual temperature is shown in Fig. 9. Arctic high amplification index concentrates near the North Pole in general. The Antarctic amplification always occurs in East Antarctic inland, and disappears in the Antarctic coast and West Antarctica. The polar amplification dominates the Third Pole, only except the southeast region. For Antarctica, the amplification is most extensive in MAM and JJA (not shown), and the index is lower than 2 in general. Generally, the distribution of amplification index over the Third Pole is uniform, with the value between 1 and 2. Contrary to the conspicuous Arctic amplification in DJF, majority of the region from the Third Pole fails to achieve amplification under the SSP1-2.6 socioeconomic scenario.

### Polar amplification compared with other regions under different socioeconomic scenarios

**Figure 10:**
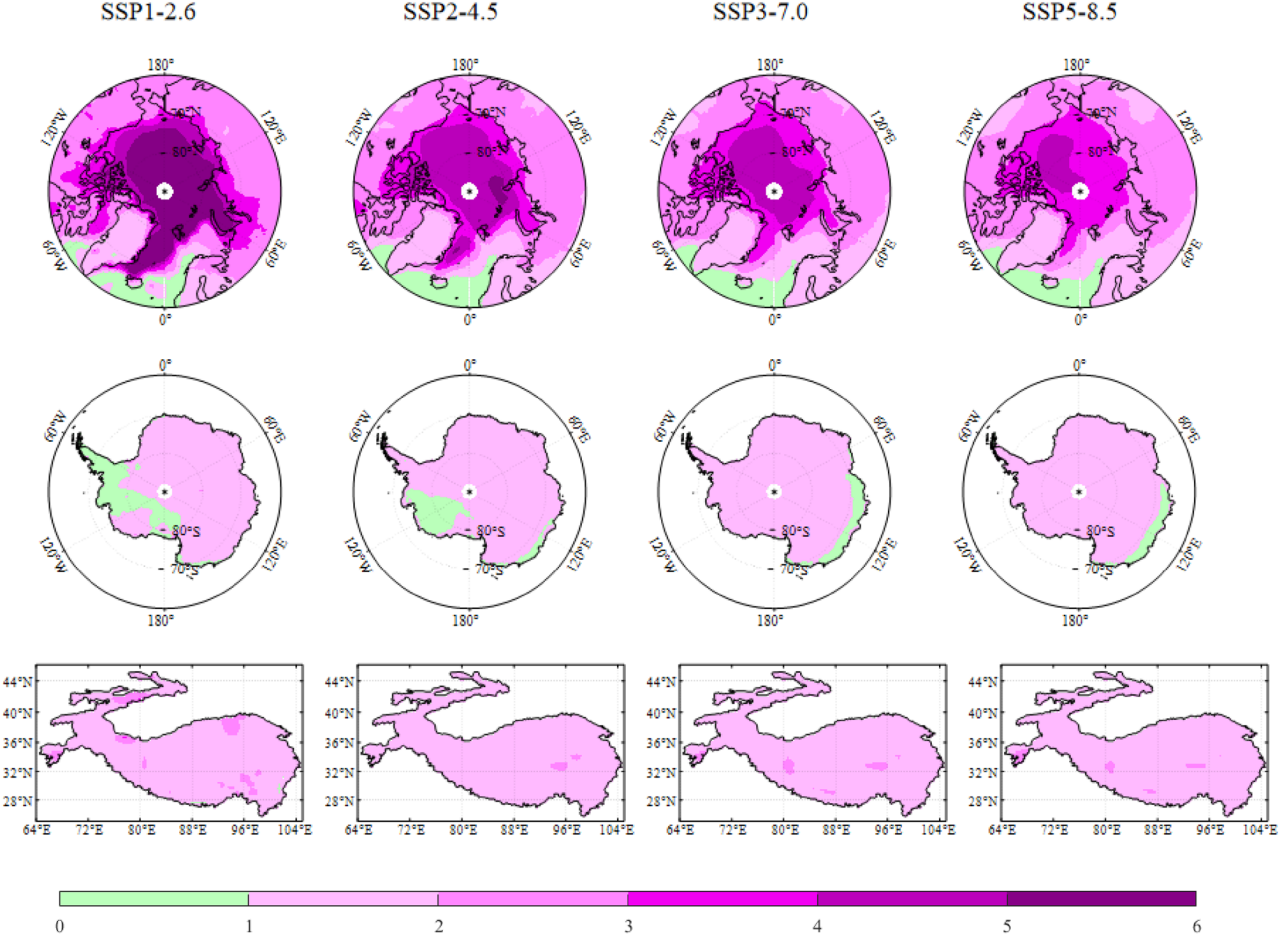
Similar to Fig. 9, but for the PAI2 that eliminating Earth’s three poles. The map was drawn in the software MATLAB R2018b (https://ww2.mathworks.cn/products/matlab.html).

The above research suggests that Earth’s three poles generally warm faster than the global mean warming. To further explore the polar amplification compared with the other regions, this study has also defined one other new polar amplification index (PAI2), as the ratio of the SAT trend over Earth’s three poles and that over other regions except the Earth’s three poles, which can eliminate the self-influence from the Earth’s three poles. Table 2 lists the new index, and Fig. 10 shows the corresponding spatial patterns for the three poles amplification, respectively.

**Table 2:** Similar to Table 1. but for the Amplification Index eliminating Earth’s three poles mean (PAI2).

	Annual	MAM	JJA	SON	DJF
**Arctic**					
SSP1-2.6	4.29	3.60	1.90	5.28	6.32
SSP2-4.5	3.14	2.79	1.54	3.55	4.65
SSP3-7.0	2.96	2.69	1.58	3.16	4.39
SSP5-8.5	2.78	2.63	1.60	2.81	4.08
**Antarctica**					
SSP1-2.6	1.37	1.76	0.91	1.39	1.41
SSP2-4.5	1.31	1.38	1.34	1.18	1.34
SSP3-7.0	1.30	1.54	1.25	1.13	1.27
SSP5-8.5	1.36	1.57	1.37	1.18	1.34
**The Third Pole**					
SSP1-2.6	1.66	1.77	1.80	1.86	1.24
SSP2-4.5	1.58	1.42	1.51	1.76	1.61
SSP3-7.0	1.70	1.74	1.56	1.71	1.79
SSP5-8.5	1.72	1.76	1.57	1.81	1.76

Compared with PAI1, PAI2 shows that the PA obviously enhances more sharply from Fig. 10 and Table 2 than that from Fig. 9 and Table 1, and the polar warming is conspicuously faster than the other regions of global mean under all SSPs. Especially for Arctic in DJF, the AA index is more than 4 and even up to 6. Even in Antarctica, almost all the indexes are above 1.0, only except for austral winter in SSP1-2.6 scenario. Similar to the previous index PAI1 from Table 1, the AA from Table 2 is the most conspicuous among the Earth’s three poles, and the strongest AA occurs in winter, with all PAI2 index over the Arctic Ocean almost more than 4 in DJF. The weakest AA is in JJA with the PAI2 of 1.90 in SSP1-2.6. In general, Arctic is from 1.5 to 6.3 times the global mean after eliminating Earth’s three poles mean warming. The AA becomes weaker from SSP1-2.6 to SSP5-8.5, with exception of JJA. Differently from PAI1, PAI2 illustrates that the AnA index (Table 2) displays the obvious seasonal variations, and increases highest in austral autumn, with index values of 1.76, 1.38, 1.54 and 1.57 in SSP1-2.6, SSP2-4.5, SSP3-7.0 and SSP5-8.5, respectively. However, the coastal of the East Antarctica always miss the amplification signal in austral summer DJF in all SSPs (not shown). In addition, Antarctic Peninsula rarely illustrates the amplification in austral spring SON and summer DJF, with exception of the SSP5-8.5. Similar to PAI1, PAI2 shows that the most conspicuous warming occurs in autumn SON over the Third Pole, with the PAI2 of 1.86, 1.76, 1.71 and 1.81 under SSP1-2.6, SSP2-4.5, SSP3-7.0 and SSP5-8.5, respectively. The amplification dominates the Third Pole in all socioeconomic scenarios, and becomes much stronger in boreal autumn MAM and winter DJF. Similar to the PAI1, PAI2 also demonstrates and exaggerates that the intensity of AA generally decreases from SSP1-2.6 to SSP5-8.5, whereas the amplification over Antarctica and the Third Pole has a distinct accelerating tendency with the increase of forcing.

## Discussions

The climate sensitivity and inter-model spread of the past climate simulations may introduce uncertainty into the projection of future climate. Compared to CMIP5, the CMIP6 models show a higher average climate sensitivity, and the CMIP6 models tend to overestimate the trend of global mean surface temperature^11, 42^. In addition, we calculate the trend using the simple linear regression, but not consider serial correlation effect of the temperature time series, and the trends should be narrower than the real expected values^43^. Most climate models fail to represent the amplitude of polar temperature change observed in paleoclimatic archives, which relates to the underestimation of aerosols effect on polar regions, and this bias may influence future predictions^44^. Surface albedo, water vapor feedbacks, atmospheric and oceanic heat transport are important processes to the warming of polar regions^45, 46^. Previous research has found that the ice-albedo feedback can be influenced by clouds, and one third of the models in CMIP6 refer to the cloud feedbacks inaccuracy, and the feedback strength depends on a large number of physical processes and parametrizations, which differs remarkably among the models^33^. Compared with CMIP5, CMIP6 has reduced the models range, and shows better performance in representing the distribution of sea ice concentration^47^. The Greenland Ice Sheet mass loss in CMIP6 equates to a CMIP5 scenario with twice the global radiative forcing^10^. Antarctic sea ice extent (SIE) has always been underestimated in austral summer^34^. The CMIP6 MMEM can perform the seasonal cycles of the Arctic and Antarctic SIE, while it fails to represent the faster decline of Arctic SIE and the larger interannual variability in Antarctic SIE after 2000^48^. CMIP6 MMEM can capture the climatological spatial distribution of Amundsen Sea Low, but underestimates the intensity^49^. Over the Third Pole, CMIP6 models exhibit a warm temperature bias in summer and cold bias in winter^50^. Compared with the CMIP5 models, CMIP6 models perform generally better in the Third Pole^51^. The spring sensible heating over the Third Pole has important influence on the Asian summer monsoon, and the future outputs from CMIP6 confirms an increasing trend, and gets stronger with the increase of forcing from SSP1-2.6 to SSP5-8.5^52^.

Under different global warming thresholds, Arctic and Antarctica are both sensitive to the increase in atmospheric CO_2_ concentration, and the strong warming occurs in Arctic in all socioeconomic scenarios^53^. Similarly, the strong increase of Arctic temperature can be observed in all cases when global warming exceeds from 1.5 °C/2.0°C to 2.0 °C/3.0°C thresholds. For Antarctica, temperature increases basically consistent with the global mean under the 1.5 °C and 2.0 °C thresholds, whereas the strong local warming over Antarctica occurs when global warming exceeds 1.5 °C threshold^49^. Consistently, the increase of temperature higher than 0.5 °C can be captured in some regions over East Antarctica when global mean warms from 1.5 °C to 2.0 °C threshold. For the Third Pole, it will achieve the 1.5 °C and 2.0 °C warming approximately 10 years earlier than the global scale^54^. When global temperature rises by 0.5 °C, the Third Pole exhibits the stronger warming in all socioeconomic scenarios.

The fastest Arctic warming occurs in DJF under all socioeconomic scenarios, which is identical with the previous estimation with CMIP5, which holds the point that Arctic warming is most pronounced in winter and autumn^55^. Corresponding to it, the strongest AA appears in these seasons, whereas the sign is weaker in summer^5, 56^, and the characteristics will exist in the near future in all SSPs. The mechanism of AA can be divided into local and remote forcing, and they may interact and boost each other^24, 57^. Sea ice plays an important role in AA, and the greater absorption of solar radiation in summer can promote anomalous latent and sensible heat fluxes in the autumn. These factors delay sea ice growth in cold seasons, induce the warmer and moister Arctic air masses into Arctic, and accelerate the conspicuous AA in autumn and winter^58^. In addition, the warming cloud feedback also influences the fast warming^59^. Strong positive ice-temperature feedbacks increase the chances of further rapid warming^13^, and it indicates the greater AA may appear. In JJA, the increase of the absorbed shortwave radiation is very much dictated offset by the greater sea ice loss in Arctic^33^. The tropospheric-mean atmospheric warming and greenhouse gas increases for tropical and Arctic regions contribute to the Arctic amplification, in addition, non barotropic and surface warming sensitivity effects also accelerates the amplification^44^. However, CMIP5 models hold the point that changes in downward longwave radiation flux from a cloudless atmosphere is the major factor affecting the AA^60^. Research on the future AA provides that ocean coupling is responsible for AA during the twenty-first century, mostly via thermodynamic coupling^61^.

The projected changes over the coastal areas of Antarctica do not match linearly with global forcing. Compared with the high forcing, the warming over the coastal Antarctica is stronger in the low forcing relative to the global mean^62^. In SSP3-7.0 and SSP5-8.5, the warming over the East Antarctica ice sheet (EAIS) is conspicuous. EAIS is highly sensitive to ocean forcing for the geometries and connectivity to the Southern Ocean, and the southerly shifts of Southern Hemisphere Westerlies may induce the general warming^63^. EAIS performs a dynamic response to the continued anthropogenic warming, and its contribution to future global sea-level may be underestimated^64^, and it is more obvious reflected in the conspicuous warming signal in CMIP6. When different RCP scenarios and thresholds are selected, there are great differences between atmosphere and sea ice, and keeping global warming below 1.5°C has a significant impact on the distribution of sea ice in the Southern Ocean^65^. Over Antarctica, the greatest amplification occurs in austral autumn MAM. Increased poleward moisture transport is the largest contributor to projected Antarctic warming, and seasonal ocean heat storage contribute a winter peak^66^, and it relates to the weaker AnA in winter than in autumn. Among the Earth’s three poles, Antarctic amplification is the weakest in general. Firstly, Southern Ocean is the crucial factor for the huge accumulated ocean heat uptake^7^. Secondly, the high elevation of Antarctica plays an important role in decreasing the susceptibility to CO_2_-forced warming^67^.

In recent decades, especially at the beginning of the twenty-first century, the Tibetan Plateau (TP) has been experiencing a more rapid warming than its surrounding regions^68^. The CMIP5 MMEM has found that the warming amplification in high altitude regions of the northern hemisphere is greater in higher greenhouse gas emission scenario^69^. The amplification of the Third Pole is conspicuous in the different scenarios in CMIP5, and the warming rate might make an obvious discrepancy to the future amplification at different thresholds^20^. The Third Pole amplification continues to exist in CMIP6, and becomes stronger in the higher forcing scenario. Altitude and latitude both contribute to the warming of the Third Pole, and the snow albedo feedback also plays an important role in the Third Pole warming amplification^70^. In addition, the enhancement of downward clear-sky longwave radiative fluxes and surface albedo feedback also promote the accelerated warming^71^. So far, there is no evidence that the Third Pole amplification relates to global circulation changes, and the mechanisms focus on the local to the mountain ranges^27^.

## Conclusions

This study provides the initial evaluation on amplification comparison of Earth’s three poles surface temperature variations from 1850 to 2100 based on the CMIP6 multi-model outputs under five socioeconomic pathways of SSP1-1.9, SSP1-2.6, SSP2-4.5, SSP3-7.0 and SSP5-8.5. The main results are summarized as the following.

(1). Among Earth’s three poles, Arctic displays the most conspicuous warming, followed by the Third Pole, and Antarctica generally shows the weakest warming. The warming trend over Earth’s three poles will enhance with the increase of forcing from low emissions scenario SSP1-1.9 to high emissions scenario SSP5-8.5.

(2). Arctic shows the strongest warming in winter, The Third Pole shows the most conspicuous in autumn. Similarly to Arctic, the Antarctic temperature exhibits the characteristic of the highest warming in austral winter. Under SSP5-8.5, the warming trend is 1.55 °C, 0.84 °C and 0.65 °C per decade over Arctic, The Third Pole and Antarctica, respectively.

(3). When global warming exceeds from 1.5 °C to 2.0 °C thresholds, Arctic annual temperature increases higher than 0.85 °C under all socioeconomic scenarios. The Third Pole also warms higher than the global mean, but lower than Arctic except in summer. Differently, Antarctica warms always lower than 0.5 °C except in SSP1-2.6.

(4). When global warming of 2.0 °C to 3.0 °C, the increase of Arctic annual temperature exceeds 2.3 °C, with the warming more than 3.0 °C in winter under all scenarios. However, Antarctica fails to warm 1.0 °C in most cases. The Third Pole also follows Arctic with the annual warming higher than 1.3 °C.

(5). AA always occurs, with the weakest amplification (PAI1 > 1.34) in summer, and the strongest (PAI1 > 2.73) in winter under different socioeconomic scenarios. For the Third Pole, the amplification is strong in summer (PAI1 > 1.31) and autumn (PAI1 > 1.09). AnA only occurs in SSP5-8.5, with the annual PAI1 of 1.03. In SSP3-7.0 and SSP5-8.5, the slightly AnA occurs in austral autumn(PAI1 > 1.16) and winter (PAI1 > 1.09). The AA intensity becomes weak with the increase of forcing, whereas the Antarctica and the Third Pole show the reverse phenomenon. This demonstrates that the rate of future warming might make a large difference from nowadays among Earth’s three poles under different scenarios.

(6). To eliminate the self-influence from Earth’s three poles warming, this study has especially defined one other new PA index (PAI2). The result shows that the amplification enhances obviously over Earth’s three poles under different scenarios, especially for Arctic in winter (DJF, PAI2 > 4.08), and even for Antarctica in austral autumn (PAI2 > 1.38). The PA warming over the Earth’s three poles is conspicuously faster than the other regions of global mean through the whole years in all the scenarios.

This study demonstrates that the rate of future warming might make a large difference from nowadays among Earth’s three poles under different scenarios and/or at different thresholds. Our findings call for the separate contribution from natural variability and anthropogenic forcing. Understanding any differences therein is of vital importance for future adaptation and mitigation strategies over the regions. Despite the greatly improved understanding of spatial and temporal variations of polar amplification, large uncertainties still exist in additional areas of research that require further investigation, especially for Antarctic amplification with scarce research. The uncertainties in polar amplification highlight the importance of further improving the atmosphere and sea ice/snow dynamical and parameterization processes in the state-of-the-art models. The new models will be released in succession, and the more models can be conductive to further improve the estimate accuracy, and to understand well the amplification mechanism over Earth’s three poles. Future research needs to further explore the PA vertical profiles with remote sensing data, and to investigate the variations of temperature extremes over Earth’s three poles under different future scenarios, both for better scientific understanding the role of polar amplification playing in climate change and for policy making.

## Supplementary Information

Supplementary Information.

## Data Availability

Publicly available datasets used in this study are from the CMIP6 website https://esgf-node.llnl.gov/projects/cmip6/.
