# Supplementary material for: Polar amplification comparison among Earth’s three poles under different socioeconomic scenarios from CMIP6 surface air temperature
Source: Sci Rep. 2022 Oct 3;12:16548. doi: 10.1038/s41598-022-21060-3 (PMC9529914; doi:10.1038/s41598-022-21060-3)
Supplement: Supplementary file 1 — Supplementary Information. [file 41598_2022_21060_MOESM1_ESM.docx]

Table S1. The annual and seasonal mean temperature (℃) for Arctic, Antarctica and the Third Pole when global warming reaches the different thresholds of 2.0 ℃ and 1.5 ℃ under different SSPs of SSP1-2.6, SSP2-4.5, SSP3-7.0 and SSP5-8.5.

| Arctic | | | | | |
| --- | --- | --- | --- | --- | --- |
|  | Annual | MAM | JJA | SON | DJF |
| SSP1-2.6 | 1.07 | 0.92 | 0.34 | 1.47 | 1.54 |
| SSP2-4.5 | 0.85 | 0.64 | 0.33 | 1.18 | 1.26 |
| SSP3-7.0 | 0.92 | 0.81 | 0.39 | 1.21 | 1.25 |
| SSP5-8.5 | 1.08 | 0.99 | 0.49 | 1.43 | 1.42 |
| Antarctica | | | | | |
| SSP1-2.6 | 0.55 | 0.58 | 0.46 | 0.38 | 0.79 |
| SSP2-4.5 | 0.22 | 0.11 | 0.36 | 0.18 | 0.22 |
| SSP3-7.0 | 0.34 | 0.42 | 0.43 | 0.16 | 0.37 |
| SSP5-8.5 | 0.31 | 0.21 | 0.41 | 0.18 | 0.43 |
| The Third Pole | | | | | |
| SSP1-2.6 | 0.78 | 0.77 | 0.82 | 0.84 | 0.71 |
| SSP2-4.5 | 0.61 | 0.63 | 0.50 | 0.64 | 0.69 |
| SSP3-7.0 | 0.52 | 0.48 | 0.49 | 0.55 | 0.57 |
| SSP5-8.5 | 0.70 | 0.51 | 0.79 | 0.88 | 0.61 |

Note: The SSP1-1.9 is omit here, for the global warming fails to achieve the 2.0 ℃ threshold.

Table S2. Similar to table S1, but for the differences between 3.0 ℃ and 2.0 ℃ threshold.

| Arctic | | | | | |
| --- | --- | --- | --- | --- | --- |
|  | Annual | MAM | JJA | SON | DJF |
| SSP2-4.5 | 2.45 | 2.11 | 1.17 | 2.89 | 3.65 |
| SSP3-7.0 | 2.70 | 2.30 | 1.26 | 3.30 | 3.93 |
| SSP5-8.5 | 2.37 | 1.94 | 1.20 | 2.91 | 3.45 |
| Antarctica | | | | | |
| SSP2-4.5 | 0.98 | 0.94 | 0.88 | 0.94 | 1.13 |
| SSP3-7.0 | 0.97 | 0.86 | 0.88 | 1.12 | 1.00 |
| SSP5-8.5 | 0.93 | 1.16 | 1.06 | 0.66 | 0.86 |
| The Third Pole | | | | | |
| SSP2-4.5 | 1.39 | 1.20 | 1.38 | 1.62 | 1.34 |
| SSP3-7.0 | 1.48 | 1.48 | 1.46 | 1.55 | 1.44 |
| SSP5-8.5 | 1.60 | 1.83 | 1.41 | 1.56 | 1.62 |

Table S3. List of CMIP6 models used in this study.

| Model Number | Model centre | Model name | Lat x Lon grid |
| --- | --- | --- | --- |
| 1 | CAS | FGOALS-g3 | 80 x 180 |
| 2 | CCCma | CanESM5 | 64 × 128 |
| 3 | EC-Earth-Consortium | EC-Earth3-Veg | 256 × 512 |
| 4 | IPSL | IPSL-CM6A-LR | 143 × 144 |
| 5 | MIROC | MIROC6 | 128 × 256 |
| 6 | MRI | MRI-ESM2-0 | 160 x 320 |
| 7 | NOAA-GFDL | GFDL-ESM4 | 180 x 288 |
